# Supplementary material for: Statistical population reconstruction of moose (Alces alces) in northeastern Minnesota using integrated population models
Source: PLoS One. 2022 Sep 27;17(9):e0270615. doi: 10.1371/journal.pone.0270615 (PMC9514631; doi:10.1371/journal.pone.0270615)
Supplement: Attachment — Submitted filename: Response to Reviewers.docx [file pone.0270615.s001.docx]

PONE-D-22-16773

Statistical Population Reconstruction of Moose (*Alces alces*) in Northeastern Minnesota using Integrated Population Models

PLOS ONE

Dear Dr. Severud,

Thank you for submitting your manuscript to PLOS ONE. After careful consideration, we feel that it has merit but does not fully meet PLOS ONE’s publication criteria as it currently stands. Therefore, we invite you to submit a revised version of the manuscript that addresses the points raised during the review process.

We received two sets of reviews. Both are very positive about the manuscript. However, the reviewers make many suggested revisions, which are mostly minor, but important for improving the manuscript.

Please submit your revised manuscript by Sep 08 2022 11:59PM. If you will need more time than this to complete your revisions, please reply to this message or contact the journal office at plosone@plos.org. When you're ready to submit your revision, log on to https://www.editorialmanager.com/pone/ and select the 'Submissions Needing Revision' folder to locate your manuscript file.

Please include the following items when submitting your revised manuscript:

A rebuttal letter that responds to each point raised by the academic editor and reviewer(s). You should upload this letter as a separate file labeled 'Response to Reviewers'.

A marked-up copy of your manuscript that highlights changes made to the original version. You should upload this as a separate file labeled 'Revised Manuscript with Track Changes'.

An unmarked version of your revised paper without tracked changes. You should upload this as a separate file labeled 'Manuscript'.

If you would like to make changes to your financial disclosure, please include your updated statement in your cover letter. Guidelines for resubmitting your figure files are available below the reviewer comments at the end of this letter.

If applicable, we recommend that you deposit your laboratory protocols in protocols.io to enhance the reproducibility of your results. Protocols.io assigns your protocol its own identifier (DOI) so that it can be cited independently in the future. For instructions see: https://journals.plos.org/plosone/s/submission-guidelines#loc-laboratory-protocols. Additionally, PLOS ONE offers an option for publishing peer-reviewed Lab Protocol articles, which describe protocols hosted on protocols.io. Read more information on sharing protocols at https://plos.org/protocols?utm_medium=editorial-email&utm_source=authorletters&utm_campaign=protocols.

We look forward to receiving your revised manuscript.

Kind regards,

Masami Fujiwara, PhD

Academic Editor

PLOS ONE

Journal Requirements:

When submitting your revision, we need you to address these additional requirements.

1. Please ensure that your manuscript meets PLOS ONE's style requirements, including those for file naming. The PLOS ONE style templates can be found at

https://journals.plos.org/plosone/s/file?id=wjVg/PLOSOne_formatting_sample_main_body.pdf and

https://journals.plos.org/plosone/s/file?id=ba62/PLOSOne_formatting_sample_title_authors_affiliations.pdf

2. We note that the grant information you provided in the ‘Funding Information’ and ‘Financial Disclosure’ sections do not match.

When you resubmit, please ensure that you provide the correct grant numbers for the awards you received for your study in the ‘Funding Information’ section.

***We apologize for the error in the funding statement. We have reconciled the documents. We have provided grant numbers where available; some funding sources do not use grant numbers.***

3. Thank you for stating the following in the Acknowledgments Section of your manuscript:

"This material is based upon work supported in part by the Center for Applied Mathematics (CAM) Summer Research Program at the University of St. Thomas. We thank T. Arnold for inspiring this work, J. Fieberg for providing parameters from Lenarz et al. (2010), the MNDNR Wildlife Health Program, and dozens of volunteers and technicians for all moose capture and monitoring work. Collaring work was funded by Voyageurs National Park, a grant from the USGS-NPS Natural Resource Preservation Program, University of Minnesota-Duluth, U.S. Fish and Wildlife Service Tribal Wildlife Grant, U.S. Environmental Protection Agency Great Lakes Restoration Initiative, and the Bureau of Indian Affairs Endangered Species Program, Minnesota Zoo Ulysses S. Seal Conservation Fund, Indianapolis Zoo Conservation Fund."

We note that you have provided funding information that is not currently declared in your Funding Statement. However, funding information should not appear in the Acknowledgments section or other areas of your manuscript. We will only publish funding information present in the Funding Statement section of the online submission form.

Please remove any funding-related text from the manuscript and let us know how you would like to update your Funding Statement. Currently, your Funding Statement reads as follows:

"CAE was financially supported in part by the Center for Applied Mathematics Summer Research Program at the University of Saint Thomas (https://cas.stthomas.edu/departments/areas-of-study/mathematics/center-for-applied-mathematics/). Moose collaring work was funded by Voyageurs National Park (https://www.nps.gov/voya/index.htm; SKW and RAM), the USGS-NPS Natural Resource Preservation Program (PMIS 140435; SKW and RAM), University of Minnesota-Duluth (https://nrri.umn.edu/; SKW and RAM), U.S. Fish and Wildlife Service Tribal Wildlife Grant (https://www.fws.gov/service/tribal-wildlife-grants; SAM), U.S. Environmental Protection Agency Great Lakes Restoration Initiative (https://www.glri.us/node/443, SAM), and the Bureau of Indian Affairs Endangered Species Program (https://www.bia.gov/bia/ots/division-natural-resources/branch-fish-wildlife-recreation/endangered-species-program; SAM), Minnesota Zoo Ulysses S. Seal Conservation Fund (https://mnzoo.org/conservation/around-world/ulysses-s-seal-conservation-grant-program/; TMW), Indianapolis Zoo Conservation Fund (https://www.indianapoliszoo.com/conservation/field-support/; TMW). The funders had no role in study design, data collection and analysis, decision to publish, or preparation of the manuscript."

Please include your amended statements within your cover letter; we will change the online submission form on your behalf.

***Thank you. We have included the amended statement in the cover letter.***

4. Please review your reference list to ensure that it is complete and correct. If you have cited papers that have been retracted, please include the rationale for doing so in the manuscript text, or remove these references and replace them with relevant current references. Any changes to the reference list should be mentioned in the rebuttal letter that accompanies your revised manuscript. If you need to cite a retracted article, indicate the article’s retracted status in the References list and also include a citation and full reference for the retraction notice.

***N/A.***

[Note: HTML markup is below. Please do not edit.]

Reviewers' comments:

Reviewer's Responses to Questions

Comments to the Author

1. Is the manuscript technically sound, and do the data support the conclusions?

The manuscript must describe a technically sound piece of scientific research with data that supports the conclusions. Experiments must have been conducted rigorously, with appropriate controls, replication, and sample sizes. The conclusions must be drawn appropriately based on the data presented.

Reviewer #1: Yes

Reviewer #2: Yes

2. Has the statistical analysis been performed appropriately and rigorously?

Reviewer #1: Yes

Reviewer #2: Yes

3. Have the authors made all data underlying the findings in their manuscript fully available?

The PLOS Data policy requires authors to make all data underlying the findings described in their manuscript fully available without restriction, with rare exception (please refer to the Data Availability Statement in the manuscript PDF file). The data should be provided as part of the manuscript or its supporting information, or deposited to a public repository. For example, in addition to summary statistics, the data points behind means, medians and variance measures should be available. If there are restrictions on publicly sharing data—e.g. participant privacy or use of data from a third party—those must be specified.

Reviewer #1: Yes

Reviewer #2: Yes

4. Is the manuscript presented in an intelligible fashion and written in standard English?

PLOS ONE does not copyedit accepted manuscripts, so the language in submitted articles must be clear, correct, and unambiguous. Any typographical or grammatical errors should be corrected at revision, so please note any specific errors here.

Reviewer #1: Yes

Reviewer #2: Yes

5. Review Comments to the Author

Please use the space provided to explain your answers to the questions above. You may also include additional comments for the author, including concerns about dual publication, research ethics, or publication ethics. (Please upload your review as an attachment if it exceeds 20,000 characters)

Reviewer #1: I thought this was an interesting study about moose populations in NE Minnesota. It was well-written and easy to read. The statistical analysis appeared to be conducted appropriately and rigorously. I do not know if the authors made all data underlying the findings of their manuscript fully available, but I selected "yes" for lack of a [*sic*] appropriate category. I have only a few minor comments, clarifications, and edits listed below.

***Thank you!***

Additional Comments to Authors.

Line 112: Add “VNP” after Voyagers National Park?

***We have added the suggested text.***

Lines 113-114: “1992-1971”? Is this a typo? If not, seems weird to list the years going backwards.

***We corrected the typo to read “***1922 ***– 1971.”***

Lines 122, 128: Change “This region” to “Our study area” to avoid ambiguity with “This”

***We have added the suggested text.***

Line 130: what does “relatively very few” mean? It could mean anything.

***We have added more quantitative detail here, “Adult and calf moose hair was present in relatively few wolf scats from VNP (0–4% occurrence) compared to scats from primary moose range in MN (7–22% occurrence).***

Line 138: “has” to “have”?

***We have added the suggested text.***

Line 193: “Previous reconstructions have then used an age-at-harvest matrix to represent each cohort”. I’m not clear what the age-at-harvest matrix looks like or how it’s implemented. The citations provided don’t appear to help understand this; they are focused on harvest reports. Also, finding the references for 31 and 32 was not trivial using a google search.

***We added citations in the appropriate place, “Previous reconstructions have then used an age-at-harvest matrix to represent each cohort [48-50]…” We agree that tribal harvest reports are often difficult to locate, but we feel they are important to highlight in this case.***

Line 205: your objective function only scores model predictions and does not account for model complexity. Won’t this by default always select the model with the most flexibility (i.e., the most complex model)?

***We did not compare competing models in this reconstruction; had we produced multiple different reconstructions, the most complex model would indeed have the lowest chi-square value. However, this would not necessarily mean that the most complex model would be selected – it would only be selected if the difference in chi-square values between it and a simpler model was significant. Whereas AIC penalizes complexity within the calculation of the metric itself, a chi-square approach penalizes complexity outside of the calculation when the models are actually compared.***

Line 254: Shouldn’t the upper limit be 1 instead of 0.929 due to years 2010 and 2013 when no moose died? Or are you excluding these years because variability was 0?

***We excluded those years because there was no variability, but have now edited the upper limit to be 1.00 based on the reviewer’s comment.***

Line 259: Same as previous comment. Shouldn’t 0.931 be 1.0 due to 2012?

***We excluded that year because there was no variability, but have now edited the upper limit to be 1.00 based on the reviewer’s comment.***

Lines 303-308: Bias variance trade-off: Does your increase in precision affect accuracy of the estimator? Is the estimator unbiased?

***Previous Monte Carlo simulation studies have demonstrated that statistical reconstruction provides an unbiased (albeit sometimes rather imprecise) estimate of population abundance; as such, we do not believe that the increase in precision affects the accuracy of our estimates. We have cited this in the text on Line 215.***

Discussion: I would have liked to see a sentence or two about life-history strategies of moose in light of your survival and fecundity estimates. If moose have a slow-paced life-history strategy, it is not surprising that reduced adult survival really affected the population. Are these periods of reduced survival rates seen by adults that greatly affected the population (2009-2013) common in other animals with similar life-history strategies? How have other populations with similar population declines and life-history strategies responded? That is, is there hope that the NE MN moose population will recover to estimates in the 8-thousands? Or will they be in the 3-thousands until another episode of reduced adult survival drops abundance even lower?

***We have added additional text in the Discussion to illustrate this point:***

***“Moose population dynamics, like those of many other large herbivores, are more impacted by variation in adult survival compared to juvenile survival [62,63]. Adult survival typically varies little [63], but*** ***in populations exhibiting low and variable adult survival, populations decline [64]. Conversely, increases in adult survival can improve population performance [65,66].”***

Similarly, without knowing too much about moose life-history strategies, I was surprised there was no detectible difference in survival between sexes. Is this consistent with other moose studies? Is not being able to detect a difference due to lack of precision in the data/estimators? In the taxa I work with, females tend to have lower survival than males due to the cost of reproduction. In summary, why didn’t you find a difference in survival of the sexes? Was it because a difference doesn’t exist with moose, or is it because the data/esitimators [*sic*] were insufficiently precise to detect a difference if a difference existed?

***There is a combination of studies not detecting a difference in survival based on sex (Lenarz et al. 2009, 2010), the ratio of collared moose being skewed to females (Grand Portage and Voyageurs), and sex differences not being reported (Carstensen et al.). Other studies of moose that have investigated sex differences in natural mortality have also found no difference by sex (Ericsson and Wallin 2001), but speculated that adult female moose face a delayed cost of reproduction that does not impact them until much older. This has now been clarified in the text.***

Finally, there is some talk about cause-specific mortality regarding parasites and disease. However, it is not clear to me what the causes of mortality were between 2009-2013 that cut the population in half. Some more expert opinion/speculation of the causes of mortality and the relative effect of each on survival would be welcome in my opinion.

***We added more clarity in the discussion to this point:***

***“The two lowest collared moose survival rates measured on the Grand Portage Indian Reservation also occurred during this time period. Additionally, opportunistically collected free-ranging moose that were necropsied showed health and disease issues were common during this same period [39]. Subsequent research on cause-specific mortality of adult moose in northeastern Minnesota further highlighted the significant effect of disease and parasites, such as winter tick and meningeal worm, on adult moose survival [24,26,39]. Additionally, wolf populations may have been subsidized by white-tailed deer in areas of moose range in Minnesota, leading to declines in moose numbers via apparent competition and inverse-density-dependent predation [61].”***

Reviewer #2: Dear Authors,

For me, this is an important study because 1) there is considerable concern in much of North America about local population declines of moose and your study is a key contribution that exemplifies the problem, and 2) we really need more case studies on the use of integrated population models to increase rigor and precision in wildlife management and conservation science by using all available data. Thank you for doing this work.

Overall, I had few issues with your manuscript, which is generally well written. Most were on issue of presentation and style, which I note below.

***Thank you!***

That said, my most substantive comments pertain to the population projection to 2030, which I suspect will be very important for local managers. Much more clarity on how these were conducted is needed, as are considerable cautions on the utility and ambiguity of the results. As is, I don't find this part of the manuscript useful, which is unfortunate.

I hope you find some of my comments helpful in revising your manuscript.

Sincerely,

Thomas Jung

Senior Wildlife Biologist, Government of Yukon

Adjunct Professor, University of Alberta

General Comments:

1) Mortality due to hunting of different sexes in the population projection to 2030 is not at all well described in the Methods or Results and it needs to be. I found it impossible to understand what the modeled projections pertain to – that is, were these for a hunted or unhunted populations, and if the former what levels of hunting for each sex. My concern is that the population projections are not going to be very useful for decision making without being much clearer on if and how hunting mortality was explicitly modeled.

***We have clarified in the text that the projection assumes no harvest mortality.***

2) Given that adult mortality appears to be driving the decline and population dynamics of the population, I would like to see substantial discussion of the causes of mortality in the Discussion, explicitly including hunting.

***We have clarified in the text that hunting mortality was not explicitly modeled because of the current harvest closure for state licenses (limited tribal harvest currently occurs). We have cited other work (Moen 2021) that does just that. We have also added more discussion about specific causes of mortality that may have been working in concert during the abrupt decline.***

3) I would appreciate some discussion and cautions regarding the projected population estimates for 2030, given the wide CI and that the CI for lambda overlap 1.

***We added further explanation of the wide CI and the usefulness of collaring work and other data streams to help inform population estimates.***

4) Ensure the tense is consistently in the past. It is not in much of the Methods, for example (e.g., Lines 120, 128, 131, etc). A careful revision is required.

***We have revised the manuscript for tense.***

Detailed Comments:

Line 25: The paper would have wider appeal if you could expand this to more than just Minnesota. I believe that recent declines in moose abundance are happening broadly across North America?

***We have added more context to include other populations.***

Line 28: As per above, delete “across the state”

***We have deleted the phrase.***

Line 36: Replace “ten” with “10”

***We replaced “ten” with “10.”***

Line 38. 0.689 is a really low annual survival estimate for an adult female ungulate.

***We agree and put that rate in context later in the manuscript.***

Line 41: Rephrase without saying “leading us to conclude”

***This sentence has been rephrased.***

Lines 49-55: While I appreciate the interest for including this position statement, I think it would fit better in the main text (Introduction or Study Area) or Acknowledgements. The editors may be able to provide more specific guidance.

***We respectfully disagree and, in solidarity with our tribal partners, wish to retain the statement as is.***

Line 60: Perhaps replace “parameters such as these” with “abudance [*sic*] and demographic parameters”

***We have rephrased as indicated.***

Line 69: Not only different studies, but likely more importantly is the ability of IPMs to incorporate disparate datasets from the same population (e.g., aerial surveys, radio-collared individuals, age-at-harvest, etc.).

***We have incorporated these suggestions.***

Line 74: Replace “already” with “previously”

***We have rephrased as indicated.***

Lines 88-91: Simplify to: “In response to the rapid decline of moose in northeastern Minnesota, studies of adult and calf survival and cause specific mortality were initiated (Fig. 1).”

***We incorporated some of the suggested text but retained the agency names to use active (rather than passive) voice.***

Line 92: Delete “state-of-the-art”

***We have rephrased as indicated.***

Line 98: Simplify to: “Specifically, we used statistical population reconstruction to estimate population abundance, recruitment, and estimate survival rates, using all available data.”

***We have rephrased as indicated.***

Line 104: Please note WHY you projected the estimates 10 years in the future (i.e., the management/conservation interests).

***We have clarified the text to state, “We also examined the sensitivity of model estimates to fluctuations in adult survival and per-capita recruitment (number of calves that survived to 1 year per adult female alive during the previous year) to determine which may be more important in predicting population growth and used time series analysis to project population estimates 10 years into the future to inform management and conservation concerns."***

Line 108: Rephrase to: “Our study occurred in northeastern Minnesota, near the southern limit of the distributional range of moose”

***We have rephrased as indicated.***

Line 113: Please check “1992-1971”. Should this be “1971-1992”or other? Confusing.

***We corrected the typo to read “***1922 ***– 1971.”***

Lines 113-118: This is confusing. I am unclear if there is a difference between state-licensed hunters and tribal subsistence harvest. In these years was all hunting closed, or just state-licensed hunters? Please rephrase carefully for clarity and concision. Moreover, these sentences should come closer to the end of the Study Area description.

***We clarified this in the text.***

Line 120-122: This sentence really should come after the first sentence in the first paragraph (i.e., Line 108).

***We have revised as indicated.***

Line 121: Delete: “between 47°06′N and 47°58′N latitude and 90°04′W and 92°17′W longitude”

***We deleted the coordinates.***

Line 128: Define “primary” moose range please. I have no idea what that is.

***We have rephrased for clarity.*** “***Voyageurs National Park is just west of primary moose range, which is delineated by MNDNR Section of Wildlife field and research staff (Fig. 1). Moose occur outside of primary range, but at low densities.”***

Line 131: “MN” is not indicated as Minnesota earlier in the text. Please do so.

***We have now done so at first mention.***

Line 138: Delete “has”

***We have rephrased for clarity.***

Line 139: When in winter? Provide month(s). This is relevant for classification methods if antlers were used to distinguish between males and females.

***We added the sentence: “Timing of surveys was typically during the first two weeks of January; however, insufficient snow depth postponed the 2012 survey until 26 January to 9 February [40].”***

Line 146: Change “visually identified” to “classified”. Also, briefly discuss how whtis [*sic*] was done (e.g., body size, presence of vulva patch, antlers, etc.).

***We revised the text to state: “Each sighted moose was classified as either a calf, cow, or bull based on size and presence of vulva patch and/or antlers.”***

Line 148: Avoid use of “cows and bulls”. For an international journal use adult males and adult females. Make this change throughout the text, tables, and figures, where appropriate.

***We edited this throughout.***

Lines 163-172: Simplify to: “We used annual adult survival rates from two previous studies. The first study used 150 adult moose (95 F/55 M) collared during 2002-2008 [11]; however, we only used survival rates that coincided with the aerial survey (2005–2007). We used pooled adult survival estimates because there was no difference in survival between males and females [11]. The second study was conducted from 2013-2016 and used 173 adult moose (123 F/50 M) [24]. Differences in survival between males and females were not reported, so we also used the pooled adult survival estimates from this study. Details of animal capture, handling, collaring, and monitoring can be found in the source publications [11,14,24,40].”

***We simplified to:*** ***We used annual adult survival rates from two previous studies by MNDNR, FDL, and the 1854 Treaty Authority [11,14,24,42]. The earlier MNDNR-FDL-1854 Treaty Authority study used 150 adult moose (95 F/55 M) collared during 2002–2008 [11]; however, we only used survival rates that coincided with the aerial survey (2005–2007). We used pooled adult survival estimates, because there was no difference in survival between males and females [11,14]. The more recent MNDNR study was conducted from 2013 to 2016 and used 173 adult moose (123 F/50 M) [24]. Differences in survival between males and females were not reported, so we used the pooled adult survival estimates from this study. Details of animal capture, handling, collaring, and monitoring can be found in the source publications [11,14,24,42].***

Line 173: Replace “2” with “two”

***We have edited as indicated.***

Line 193: Cite sources here please.

***We have added citations here.***

Line 197: Sure, but in Line 212 you assumed a 50:50 sex ratio of calves so you should note that here, rather than pooled in the model.

***We have additionally noted that here.***

Line 199: You need to define an “adult”

***We included the definition here.***

Line 207: use “adult females” only.

***We edited this throughout.***

Line 222: I think you mean “annual” not “yearly” survival?

***We edited this throughout.***

Line 240: What were the increments?

***We used increments of 0.1%. We have added this detail to the text.***

Line 244: Replace “1” with “one”

***We have edited as indicated.***

Line 280: How was mortality by hunting used in the population projections, if at all? This is important given the varied hunting history of moose in this region. Are these estimates without hunting?

***We have clarified in the text that our projections do not explicitly model the impact of harvest mortality on the population.***

Line 283: That’s a wide confidence interval for 2030, with a lambda CI that ranges above and below 1. Just a comment.

***We have now highlighted this in the discussion.***

Line 296: Okay. What does this mean? Are the projected estimates inclusive of harvest? This needs much better clarification as wildlife managers really need to know if and how the modeled projections include hunting and at what levels for each sex.

***Moen (2021) modeled harvest scenarios and we have cited those here. Our projections do not explicitly model harvest mortality. We have clarified that in the text.***

Line 308: Suggest change from “weather conditions” to “moose sightability”. This is because other factors such as individual behaviour or habitat use, as well as weather, can affect estimates. Moreover, aerial surveys should not be done in weather that results in substantially reduced sightability!

***We have revised this sentence for clarity.***

Line 344-357: I would suggest deleting this paragraph as it is quite tangential to the work.

***We have deleted the suggested paragraph.***

Line 358: This is not an initial assessment. Suggest replacing “initial” with “refined” or “more comprehensive”

***We replaced “initial” with “more comprehensive.”***

Line 377: Delete “such as that continuing to be undertaken by Grand Portage Band of Lake Superior Chippewa on Grand Portage Indian Reservation and in ceded territory in Superior National Forest,”

***We have retained this phrase to highlight the work of our tribal partners.***

6. PLOS authors have the option to publish the peer review history of their article (what does this mean?). If published, this will include your full peer review and any attached files.

If you choose “no”, your identity will remain anonymous but your review may still be made public.

Do you want your identity to be public for this peer review? For information about this choice, including consent withdrawal, please see our Privacy Policy.

Reviewer #1: No

Reviewer #2: Yes: Thomas Jung

[NOTE: If reviewer comments were submitted as an attachment file, they will be attached to this email and accessible via the submission site. Please log into your account, locate the manuscript record, and check for the action link "View Attachments". If this link does not appear, there are no attachment files.]

While revising your submission, please upload your figure files to the Preflight Analysis and Conversion Engine (PACE) digital diagnostic tool, https://pacev2.apexcovantage.com/. PACE helps ensure that figures meet PLOS requirements. To use PACE, you must first register as a user. Registration is free. Then, login and navigate to the UPLOAD tab, where you will find detailed instructions on how to use the tool. If you encounter any issues or have any questions when using PACE, please email PLOS at figures@plos.org. Please note that Supporting Information files do not need this step.

In compliance with data protection regulations, you may request that we remove your personal registration details at any time. (Remove my information/details). Please contact the publication office if you have any questions.
